# FABP3 Aggravates Cerebral Ischemia–Reperfusion Injury by Promoting Mitochondrial Lipid Accumulation and Enhancing BAX-Dependent Apoptosis

**DOI:** 10.3390/cells15111003

**Published:** 2026-05-29

**Authors:** Yunsi Zheng, Anqi Luo, Kohji Fukunaga, Qibing Liu, Qingyun Guo

**Affiliations:** 1 Key Laboratory of Tropical Translational Medicine of Ministry of Education, School of Basic Medical Sciences, Hainan Academy of Medical Sciences, Hainan Medical University, Haikou 571199, China; zys@muhn.edu.cn (Y.Z.); luoanqi_cpu@163.com (A.L.); 2 Department of CNS Drug Innovation, Graduate School of Pharmaceutical Sciences, Tohoku University, Sendai 980-8578, Japan; kfukunaga@tohoku.ac.jp

**Keywords:** ischemia/reperfusion, fatty acid-binding protein 3, mitochondrial dysfunction, BAX, lipid peroxidation

## Abstract

**Highlights:**

**What are the main findings?**

- FABP3 deficiency attenuates cerebral infarct injury after ischemia–reperfusion in mice and improves the neurological outcomes.
- FABP3 enhances BAX-dependent mitochondrial apoptotic signaling by promoting abnormal mitochondrial lipid accumulation and lipid peroxidation; BAX inhibition and antioxidants all partially reverse this detrimental effect.

**What are the implications of the main findings?**

- This study suggests that FABP3 may serve as an important molecular link connecting lipid metabolic imbalance, mitochondrial injury, and apoptosis in ischemic neurons.
- Targeting FABP3 and its associated lipid peroxidation–BAX pathway may provide a potential therapeutic strategy for neuroprotection in ischemic stroke.

**Abstract:**

We previously demonstrated that fatty acid-binding protein 3 (FABP3) is significantly upregulated in ischemic neurons, and its inhibition mitigates ischemic brain injury in mice and attenuates mitochondrial damage under rotenone-induced oxidative stress. These findings suggest a potential role for FABP3 in mitochondrial dysfunction in ischemic neurons, although the underlying mechanism remains unclear. In this study, we further investigated the role of FABP3 in mitochondrial injury and apoptosis in ischemic neurons. Our findings indicated that FABP3 deficiency significantly decreased infarct volume following middle cerebral artery occlusion/reperfusion (MCAO/R) in mice, improved cognitive and spontaneous activity deficits, and suppressed BAX activation and mitochondrial translocation, caspase-3 activation, and cytochrome c release. In HT22 cells subjected to oxygen-glucose deprivation/reoxygenation (OGD/R), FABP3 deficiency increased cell viability, reduced apoptosis, and alleviated the loss of mitochondrial membrane potential. Conversely, FABP3 overexpression further exacerbated mitochondrial dysfunction and apoptosis, effects that were partially reversed by the BAX inhibitor BAI1. Furthermore, FABP3 overexpression promoted abnormal mitochondrial lipid accumulation and increased lipid peroxidation. Both the mitochondria-targeted antioxidant MitoQ and the ferroptosis inhibitor Ferrostatin-1 alleviated FABP3 overexpression-induced mitochondrial damage and apoptotic signaling. Collectively, our findings suggest that FABP3 is an important promoter of cerebral ischemia–reperfusion injury. FABP3 may aggravate ischemic neuronal injury by promoting abnormal mitochondrial lipid accumulation and lipid peroxidation, thereby enhancing BAX-dependent mitochondrial apoptotic signaling. Targeting FABP3 may provide a potential therapeutic strategy for neuroprotection in ischemic stroke.

## 1. Introduction

Ischemic stroke is a major cause of death and long-term disability [1]. Although reperfusion therapy can rescue part of the ischemic penumbra, reperfusion itself also triggers a series of secondary injury responses, known as cerebral ischemia–reperfusion injury, which has become a major factor limiting further improvement in clinical outcomes [2,3]. Among the multiple mechanisms involved in reperfusion injury, mitochondrial dysfunction is considered a key determinant of neuronal survival and death [4,5,6]. During ischemia–reperfusion, the loss of mitochondrial membrane potential, excessive production of reactive oxygen species, and increased membrane permeability collectively drive cells toward irreversible injury [7,8]. Therefore, investigating the molecular mechanisms underlying mitochondrial injury is important for understanding cerebral ischemia–reperfusion injury and for identifying new neuroprotective targets [9,10].

Neurons are highly susceptible to disruptions in energy metabolism and redox balance, with mitochondria serving not only as key organelles for cellular energy production but also as a central hub for integrating apoptotic signals [11,12]. Among the various mechanisms underlying mitochondrial injury, mitochondrial-dependent apoptosis is a major pathway leading to neuronal death following ischemia [13,14,15]. Mitochondrial outer membrane permeabilization constitutes a critical event in the intrinsic apoptotic pathway, with the pro-apoptotic protein BAX identified as a major executor of this process [16]. Under resting conditions, BAX mainly exists in an inactive form in the cytoplasm. However, upon injurious stimulation, it undergoes conformational activation and translocates to mitochondria, where it oligomerizes on the outer mitochondrial membrane. This action promotes the release of pro-apoptotic factors, such as cytochrome c, into the cytoplasm, subsequently triggering the caspase cascade and leading to apoptosis [17]. Previous studies have shown that inhibiting BAX reduces neuronal apoptosis and behavioral deficits after global cerebral ischemia [18], and that BAX gene deficiency mitigates mitochondrial damage during myocardial ischemia–reperfusion [19], suggesting that BAX activation is crucial in ischemic neuronal death. Therefore, clarifying the upstream molecular mechanisms that regulate BAX activation during cerebral ischemia–reperfusion is important for understanding the key regulatory events underlying apoptosis in ischemic neurons.

In recent years, there has been a growing focus on the role of lipid metabolic imbalance in ischemic brain injury [20,21]. Abnormal lipid trafficking, increased mitochondrial lipid burden, and the consequent lipid peroxidation not only contribute to mitochondrial injury but may also directly amplify cell death signaling [22]. Fatty acid-binding protein 3 (FABP3), an intracellular lipid chaperone, binds and transports long-chain fatty acids and other hydrophobic ligands, playing an important role in maintaining cellular lipid homeostasis [23]. Our previous studies showed that FABP3 was markedly upregulated in neurons within the ischemic penumbra following cerebral ischemia–reperfusion, and that competitive interference with FABP3 ligands alleviated ischemic brain injury in mice [24]. Furthermore, previous studies have indicated a close association between FABP3 and mitochondrial injury [25,26]. These findings collectively support a detrimental role of FABP3 in ischemic neuronal injury. However, whether FABP3 merely serves as an injury-associated lipid-binding protein or actively links lipid metabolic disturbance to mitochondrial apoptotic commitment remains incompletely understood. In particular, it remains unclear whether FABP3 promotes mitochondrial apoptosis by altering mitochondrial lipid loading and inducing oxidative lipid stress.

In this study, we aimed to define further the mitochondrial mechanism by which FABP3 amplifies ischemic neuronal injury. Using in vivo and in vitro models, we investigated whether FABP3 promotes mitochondrial lipid accumulation and peroxidation, thereby facilitating BAX activation and recruitment to mitochondria, cytochrome c release, and downstream apoptosis. This work may extend previous studies by suggesting a potential FABP3–mitochondrial lipid peroxidation–BAX apoptosis axis in ischemic neurons.

## 2. Materials and Methods

### 2.1. Animals

10-week-old male wild-type (WT) C57BL/6J mice and FABP3 knockout (F3KO) mice (C57BL/6 background) were purchased from Cyagen Biosciences (Guangzhou, China). The animals were housed under controlled temperature and humidity with a 12 h light/dark cycle (9:00–21:00) and had free access to food and water. No obvious differences in general appearance, body condition, spontaneous activity, or routine feeding were observed between WT and KO mice during routine husbandry. Gross observation at the time of tissue collection did not reveal apparent abnormalities in F3KO mice. A total of 88 mice were used in this study, with four to five mice housed per cage. All mice were acclimatized to the animal facility for at least 7 days before experiments. In the acute cohort, 20 WT and 20 F3KO mice underwent ischemic surgery. In the 14-day subacute cohort, WT and F3KO mice were randomly assigned to sham groups (*n* = 10 per genotype) or ischemia–reperfusion groups (*n* = 14 per genotype). All animal procedures were performed in accordance with the Guide for the Care and Use of Laboratory Animals and received approval from the Animal Ethics Committee of Hainan Medical University (Approval No. HYLL-2023-432; Approval Date: 26 September 2023).

### 2.2. Establishment of the MCAO/R Model

A transient middle cerebral artery occlusion/reperfusion (MCAO/R) mouse model was established as previously described [27]. Briefly, under continuous isoflurane anesthesia, the right common, external, and internal carotid arteries were exposed. A silicone-coated monofilament (602156PK5Re, Doccol Corporation, Sharon, MA, USA) was inserted into the right external carotid artery and advanced through the internal carotid artery to the origin of the middle cerebral artery. After 1 h of occlusion, the filament was withdrawn to permit reperfusion. Sham-operated mice underwent the same surgical procedures, except for filament insertion. During ischemia and reperfusion, body temperature was maintained at 37 °C using a thermostatically controlled heating pad. Regional cerebral blood flow was monitored using a laser Doppler flowmeter to confirm successful ischemia, and a decrease of approximately 70–90% from baseline was considered successful model induction.

### 2.3. Evaluation of Infarct Volume

Mice were euthanized at 24 h or 14 d after reperfusion by deep isoflurane anesthesia and either by cervical dislocation or by decapitation after cardiac perfusion. Brains were rapidly removed and pre-cooled at −30 °C for 10 min. Subsequently, the brains were sectioned into five 2 mm-thick coronal slices. Infarct staining was performed by incubating the brain slices in 1% 2,3,5-triphenyltetrazolium chloride (TTC; T8877, Sigma-Aldrich, St. Louis, MO, USA) at 37 °C for 20 min, followed by overnight fixation in 4% paraformaldehyde. After TTC incubation, viable tissue was identified by its red coloration, whereas infarcted tissue appeared white. Infarct areas were quantified using the ImageJ software (version 1.54p; National Institutes of Health, Bethesda, MD, USA) and calculated as the sum of infarct area × section thickness across all five sections and expressed as a percentage of the contralateral hemisphere volume: infarct volume (%) = total infarct volume/total contralateral hemisphere volume × 100%.

### 2.4. Neurological Score

Neurological function was evaluated using a 0–4 neurological deficit scoring system. The criteria for scoring were as follows: 0, normal motor function; 1, forelimb flexion when suspended by the tail; 2, circling to the contralateral side when suspended by the tail on a flat surface, with normal resting posture; 3, spontaneous leaning to the contralateral side during free movement; and 4, absence of spontaneous movement and significantly reduced consciousness. Sham-operated mice typically exhibited no neurological deficits.

### 2.5. Y-Maze Test

The Y-maze test was performed to evaluate spatial working memory and spontaneous exploratory behavior as previously described [28]. Each mouse was placed at the terminus of one arm of the Y-maze and allowed to explore freely for 8 min. The sequence of arm entries and the total number of arm entries were recorded. Spontaneous alternation was defined as consecutive entries into three distinct arms and was calculated as follows: spontaneous alternation (%) = number of alternations/(total arm entries − 2) × 100%.

### 2.6. Rotarod Test

The rotarod test was used to assess motor coordination and balance. The apparatus consisted of a base and a rotating rod with a non-slip surface, measuring 3 cm in diameter and 30 cm in length. Before formal testing, mice were trained on the rotating rod at 20 rpm until their latency to fall exceeded 100 s. During the formal test, the latency to fall within 300 s was recorded.

### 2.7. Open Field Test

The open field test was used to evaluate spontaneous locomotor activity and exploratory behavior. Each mouse was placed in the center of an open field arena (40 cm × 40 cm × 40 cm) and allowed to explore freely. The total distance traveled, the percentage of time spent in the center zone, and the percentage of distance traveled in the center zone were recorded over 5 min. After each trial, the arena was cleaned with 75% ethanol to eliminate olfactory cues. Locomotor trajectories were quantified using the corresponding analysis software.

### 2.8. Cell Culture

HT22 mouse hippocampal neuronal cells were purchased from Procell Biotechnology (Wuhan, China) and cultured in Dulbecco’s modified Eagle medium (DMEM) supplemented with 10% fetal bovine serum and 1% penicillin-streptomycin at 37 °C in a humidified incubator containing 5% CO_2_. FABP3-deficient HT22 cells, FABP3-overexpressing lentiviral vectors (OE-F3), and the corresponding empty vector control (Vec) were purchased from GeneReal Biotechnology (Guangzhou, China). The efficiency of transfection or selection was confirmed through Western blotting and fluorescence imaging before subsequent experiments.

### 2.9. OGD/R Model and Drug Treatment

Oxygen-glucose deprivation/reoxygenation (OGD/R) was employed to replicate in vitro ischemia–reperfusion injury. HT22 cells were cultured in glucose-free DMEM and incubated in a 95% N_2_ and 5% CO_2_ atmosphere for 2 h. Subsequently, the medium was replaced with complete culture medium, and the cells were returned to normoxic conditions for 24 h of reoxygenation. To assess the roles of lipid peroxidation and mitochondrial oxidative stress, cells were pretreated 1 h before OGD with the BAX inhibitor BAI1 (10 μM, MedChemExpress, Monmouth Junction, NJ, USA, Cat. No. HY-103269), the mitochondria-targeted antioxidant mitoquinone mesylate (MitoQ, 1 μM; HY-100116A, MedChemExpress), or the ferroptosis inhibitor Ferrostatin-1 (10 μM; HY-100579, MedChemExpress). BAI1, MitoQ, and Ferrostatin-1 were first dissolved in dimethyl sulfoxide (DMSO) to prepare stock solutions, which were then diluted in culture medium to the indicated working concentrations. The final DMSO concentration was kept identical across all treatment groups, and cells treated with the same concentration served as vehicle controls. These reagents were maintained throughout the reoxygenation period.

### 2.10. Cell Viability Assay

Cell viability was assessed using the Cell Counting Kit-8 (CCK-8; 96992, Sigma-Aldrich) assay. Cells were seeded into 96-well plates, and after treatment, 10 μL of CCK-8 reagent was added to each well, then incubated at 37 °C for 2 h. The absorbance was subsequently measured at 450 nm using a microplate reader. Cell viability was quantified as a percentage relative to that of the control group.

### 2.11. TUNEL Staining

DNA fragmentation and apoptosis were detected using a TUNEL assay kit (C1088, Beyotime, Shanghai, China). Cells were fixed with 4% paraformaldehyde, permeabilized with Triton X-100, and incubated with the TUNEL reaction solution at 37 °C for 1 h, followed by nuclear counterstaining with Hoechst 33342. Images were acquired from multiple random fields, and apoptosis was quantified as the percentage of TUNEL-positive cells relative to the total number of Hoechst-positive nuclei.

### 2.12. Mitochondrial Analysis

Mitochondria were labeled using MitoTracker Red FM staining (M22425, Thermo Fisher Scientific, Waltham, MA, USA). Cells were incubated with 500 nM MitoTracker Red at 37 °C for 15 min, followed by fluorescence imaging. Tetramethylrhodamine ethyl ester perchlorate staining (TMRE; 87917, Sigma-Aldrich) was conducted to evaluate mitochondrial membrane potential. Cells were incubated with 500 nM TMRE in the dark for 30 min, after which the fluorescence intensity was recorded.

### 2.13. Isolation of Mitochondrial and Cytosolic Fractions

Mitochondrial and cytosolic fractions were isolated from tissues or cells at 4 °C using a mitochondrial isolation kit (MP-007, Invent Biotechnologies, Plymouth, MN, USA), according to the manufacturer’s instructions. Protein concentrations were determined using the bicinchoninic acid (BCA) protein assay (BL521A, Biosharp, Hefei, China). In Western blot analyses, COXIV served as a mitochondrial marker, and β-actin was used as a cytosolic marker.

### 2.14. Western Blot Analysis

Tissue or cell samples were lysed in RIPA buffer (P0013B, Beyotime) containing protease Inhibitor Cocktail (P1005, Beyotime; including 200 mM AEBSF, 30 μM Aprotinin, 13 mM Bestatin, 1.4 mM E64 and 1 mM Leupeptin in DMSO) and centrifuged at 15,000 rpm for 10 min at 4 °C. The supernatants were collected, and protein concentrations were measured using a BCA protein assay kit. Equal amounts of protein (20 μg per lane) were mixed with SDS loading buffer, denatured, separated by SDS-PAGE, and transferred onto PVDF membranes. Membranes were blocked with 5% non-fat milk for 1 h, incubated overnight at 4 °C with primary antibodies (listed in Supplementary Table S1), and then incubated with secondary antibodies for 2 h at room temperature. Protein bands were visualized using enhanced chemiluminescence (ECL) reagents (BL520A, Biosharp) and captured with a gel imaging system. Band intensity was quantified using the ImageJ software.

### 2.15. Immunofluorescence Staining

Immunofluorescence staining was performed as previously described [29]. For brain tissue samples, the brains were collected 24 h after reperfusion, fixed, dehydrated, embedded in OCT, and sectioned. The sections were blocked and incubated with primary antibodies at 4 °C for 3 d, followed by incubation with Alexa Fluor-conjugated secondary antibodies at 4 °C overnight, and the nuclei were counterstained with DAPI. For cell samples, cells were fixed with 4% paraformaldehyde for 10 min, permeabilized with 0.1% Triton X-100 for 10 min, and blocked with 3% fetal bovine serum for 30 min. Cells were incubated with primary antibodies overnight at 4 °C, followed by incubation with the appropriate Alexa Fluor-conjugated secondary antibodies for 2 h at room temperature in the dark, and the nuclei were counterstained with Hoechst 33342. For fatty acid uptake experiments, cells were incubated with 2 μM BODIPY FL C16 (green; HY-D1736, MedChemExpress) in serum-free medium before fluorescence imaging. Images were acquired with a confocal microscope and quantified using the ImageJ software.

### 2.16. Statistical Analysis

All statistical analyses were performed using GraphPad Prism 10.0 software. Data are presented as mean ± SEM. Comparisons between two groups were performed using an unpaired two-tailed Student’s *t*-test. For comparisons among multiple groups, one-way ANOVA followed by Tukey’s post hoc test was employed. In experiments involving two independent variables, a two-way ANOVA followed by Tukey’s post hoc test was applied. *p* < 0.05 was considered statistically significant.

## 3. Results

### 3.1. FABP3 Deficiency Alleviates Cerebral Infarction and Improves Cognitive and Motor Dysfunction in Ischemic Mice

**Figure 1:**
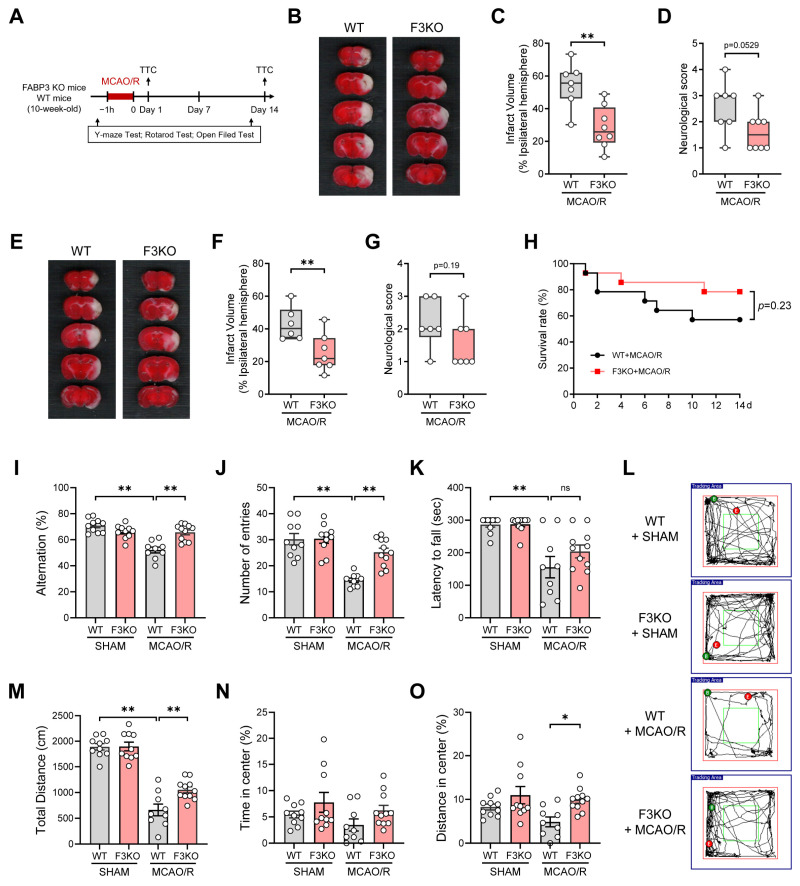
Effects of FABP3 deletion on brain injury and behavioral performance in ischemic mice. (**A**) Schematic diagram of the experimental protocol. 10-week-old wild-type (WT) and FABP3 knockout (F3KO) mice were subjected to MCAO/R or sham surgery. TTC staining was performed at 24 h and 14 days after surgery. Behavioral tests, including the Y-maze, rotarod, and open field tests, were conducted before surgery and again at 14 days after surgery. (**B**) Representative TTC-stained brain sections from WT and F3KO ischemic mice at 24 h after surgery. White regions indicate infarct areas. (**C**) Quantitative analysis of infarct volume percentage calculated from TTC staining. (**D**) Neurological deficit scores at 24 h after MCAO/R. (**E**) Representative TTC-stained brain sections from ischemic mice at 14 days after MCAO/R. (**F**) Quantitative analysis of infarct volume percentage at 14 days after MCAO/R. (**G**) Neurological deficit scores at 14 days after MCAO/R. (**H**) Survival curves of mice within 14 days after MCAO/R. (**I**,**J**) Spontaneous alternation rate (**I**) and total arm entries (**J**) in the Y-maze test. (**K**) Latency to fall in the rotarod test. (**L**–**O**) Representative movement traces (**L**), total travel distance (**M**), percentage of time spent in the center zone (**N**), and percentage of distance traveled in the center zone (**O**) in the open field test. In (**L**), the green box indicates the center zone of the open-field arena, and the letters “B” and “E” indicate the beginning and end points of the movement trajectory, respectively. Data are presented as mean ± SEM. * *p* < 0.05, ** *p* < 0.01, ns indicates no significant difference.

First, to clarify the impact of FABP3 on ischemic brain injury in mice, we established MCAO/R models in wild-type (WT) and FABP3 knockout (F3KO) mice and performed behavioral assessments before and after surgery (Figure 1A). TTC staining results showed a significant cerebral infarction in WT mice 24 h after reperfusion, whereas the infarct volume in F3KO mice was significantly reduced (Figure 1B,C). The neurological function score of F3KO mice was lower than that of WT mice (Figure 1D), suggesting that FABP3 deficiency alleviated acute ischemic brain injury. Additionally, we evaluated brain injury at 14 days post-surgery. The results revealed that the infarct volume in F3KO mice remained significantly smaller than that in WT mice (Figure 1E,F). Although the neurological function score was not statistically significant, it exhibited an improving trend (Figure 1G). Survival observation within 14 days post-surgery showed a higher survival rate in F3KO mice than in WT mice, although the difference was not statistically significant (Figure 1H). These results suggest that FABP3 deficiency effectively alleviates ischemic brain injury and may have a beneficial effect on prognosis.

Furthermore, we performed behavioral assessments of neurological function in ischemic mice. Before ischemia, WT and F3KO mice showed no significant differences in baseline behavioral performance, including cognitive, motor, and spontaneous activity (Figure S1). At 14 days post-surgery, the Y-maze test results showed that WT ischemic mice exhibited significantly lower spontaneous alternation rates and total arm entry counts compared to non-ischemic mice and F3KO ischemic mice (Figure 1I,J), suggesting that FABP3 deficiency alleviated spatial working memory impairment and improved spontaneous exploration activities after ischemia. The rotarod test demonstrated that the drop latency in WT ischemic mice was significantly shorter than that in the sham-operated group, but the difference was not statistically significant compared to F3KO ischemic mice (Figure 1K), indicating that FABP3 deficiency had a relatively limited effect on improving motor coordination and balance within 2 weeks post-surgery. Open-field tests further revealed that the total distance traveled by WT ischemic mice was significantly lower than that of the sham-operated group (Figure 1L,M), with reduced time and distance spent in the central area (Figure 1N,O), suggesting diminished spontaneous activity and a degree of anxiety-like behavior. Compared to WT ischemic mice, F3KO ischemic mice exhibited an increased total motor range and distance spent in the central region. However, no significant difference was observed in the time spent in the central region. This indicates that FABP3 deficiency has a certain ameliorative effect on post-ischemic exploratory behavior. In conclusion, FABP3 deficiency not only alleviated ischemic brain injury in mice but also improved certain cognitive functions and spontaneous activity impairments in the subacute phase following ischemia.

### 3.2. FABP3 Deficiency Inhibits Mitochondrial-Dependent Apoptosis Pathway Activation in Ischemic Mice

**Figure 2:**
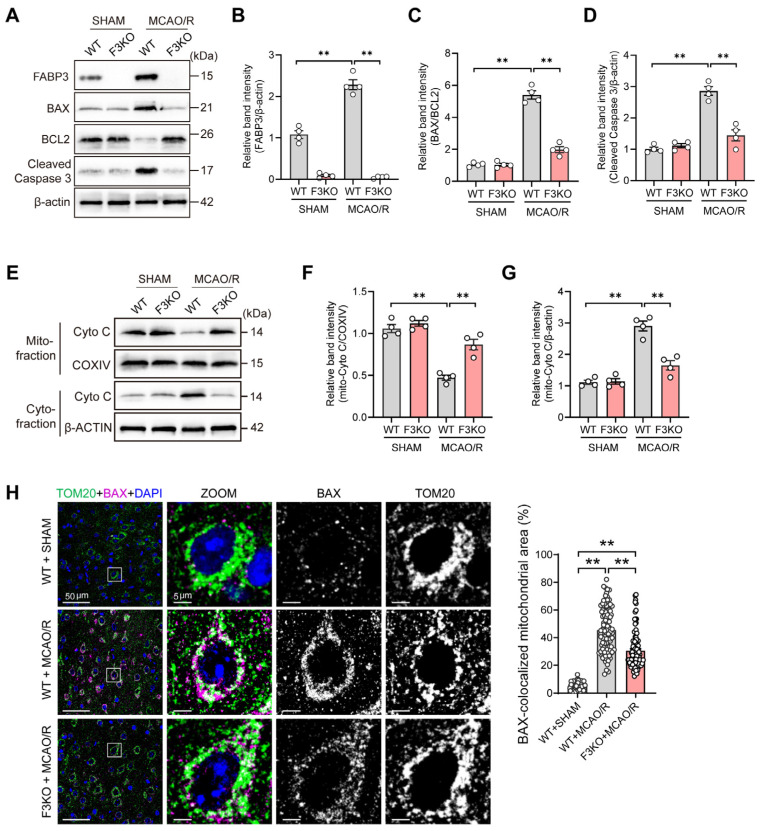
Effects of FABP3 deficiency on mitochondria-dependent apoptotic signaling in ischemic mouse brain. (**A**) Representative Western blot images of FABP3, BAX, BCL2, and cleaved caspase-3 in brain tissues from WT and F3KO mice at 24 h after sham or MCAO treatment. (**B**–**D**) Quantitative analysis of the gray value ratios for FABP3/β-actin (**B**), BAX/BCL2 (**C**), and cleaved caspase-3/β-actin (**D**). (**E**) Representative western blot images of cytochrome c in both the mitochondrial and cytosolic fractions, with COXIV serving as the mitochondrial loading control and β-actin as the cytosolic loading control. (**F**,**G**) Quantitative analysis of cytochrome c in the mitochondrial (**F**) and cytosolic (**G**) fractions. (**H**) Representative immunofluorescence images of BAX (magenta), TOM20 (green, mitochondria), and DAPI (blue, nuclei) in the cortical penumbra at 24 h after reperfusion. The small box indicates the selected region shown in the enlarged image. Scale bar = 50 μm (5 μm for the enlarged images). Quantitative analysis of the percentage of BAX and TOM20 colocalized area relative to the total TOM20-positive mitochondrial area. Data are presented as mean ± SEM. ** *p* < 0.01.

To further elucidate the impact of FABP3 deficiency on the mitochondrial apoptosis pathway during the early stages of ischemia–reperfusion, brain tissues were collected 24 h after reperfusion for protein and immunofluorescence analysis. Western blot analysis revealed that in WT mice, MCAO significantly increased the BAX/BCL2 ratio and cleaved caspase-3 levels. In contrast, in F3KO mice, this increase was significantly inhibited (Figure 2A–C), suggesting that FABP3 deficiency attenuates the activation of pro-apoptotic signals in the early ischemic phase, thereby inhibiting the mitochondrial-dependent apoptosis pathway. Concurrently, MCAO/R upregulated FABP3 expression, whereas FABP3 levels were weak in F3KO mice. Considering that BAX-mediated increased mitochondrial outer membrane permeability is a critical step in the mitochondrial apoptosis pathway, we further isolated mitochondrial and cytoplasmic components to assess changes in the subcellular distribution of cytochrome c. The results showed that cytochrome c was significantly reduced in the mitochondrial fraction of WT ischemic mice, whereas it was significantly increased in the cytoplasmic fraction, suggesting that MCAO/R induced the release of cytochrome c from mitochondria to the cytoplasm. In contrast, F3KO ischemic mice retained higher levels of cytochrome c in the mitochondrial fraction, whereas the increase in cytochrome c in the cytoplasmic fraction was smaller (Figure 2D–F), indicating that FABP3 deficiency significantly mitigates the increase in mitochondrial outer membrane permeability and subsequent cytochrome c leakage. Immunofluorescence was then employed to further examine changes in BAX localization in mitochondria. In WT ischemic mice, BAX and the mitochondrial marker protein TOM20 exhibited significant co-localization, whereas this co-localization was significantly weakened in F3KO ischemic mice (Figure 2G). Quantitative analysis confirmed that the proportion of mitochondrial area colocalized with BAX and TOM20 in F3KO ischemic mice was significantly lower than that in WT ischemic mice (Figure 2H). These results indicate that FABP3 deficiency inhibits the BAX recruitment to mitochondria following ischemia, thereby reducing the activation of the mitochondrial-dependent apoptosis cascade.

### 3.3. FABP3 Deficiency Alleviates OGD/R-Induced Mitochondrial Damage and Inhibits BAX Activation

**Figure 3:**
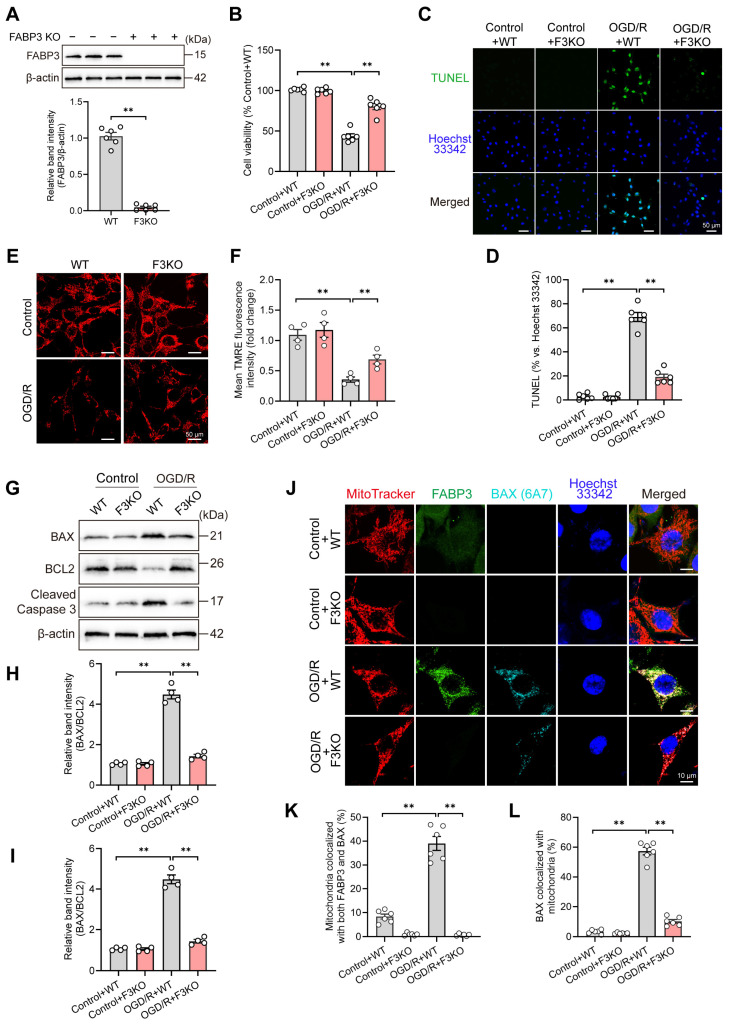
Effects of FABP3 deficiency on mitochondrial injury and BAX activation under OGD/R. (**A**) Representative Western blot images of FABP3 and quantitative analysis. (**B**) Cell viability was measured using the CCK-8 assay. (**C**,**D**) Representative TUNEL staining images (**C**) and quantitative analysis of the percentage of TUNEL-positive cells (**D**). (**E**,**F**) Representative TMRE staining images (**E**) and quantitative analysis of fluorescence intensity (**F**). (**G**–**I**) Representative western blot images of BAX, BCL2, and cleaved caspase-3 (**G**); quantitative analysis of the BAX/BCL2 (**H**) and cleaved caspase-3/β-actin (**I**) ratios. (**J**) Representative immunofluorescence images of MitoTracker (red), FABP3 (green), activated BAX (6A7, cyan), and Hoechst 33342 (nuclei, blue). In the merged images, yellow signals indicate the overlap between MitoTracker and FABP3, whereas white signals indicate the overlap among MitoTracker, FABP3, and BAX (6A7). (**K**) Quantitative analysis of the percentage of FABP3 and BAX co-localized mitochondrial area relative to the total mitochondrial area. (**L**) Quantitative analysis of the percentage of BAX-colocalized mitochondrial area relative to the total mitochondrial area. Data are presented as mean ± SEM. ** *p* < 0.01.

To verify the role of FABP3 in mitochondrial damage and apoptosis, HT22 cells were subjected to OGD/R to mimic ischemia–reperfusion-like injury. Western blot analysis revealed a significant reduction in FABP3 protein expression in F3KO cells, confirming the successful establishment of the FABP3 deficiency model (Figure 3A). Cell viability assays showed that OGD/R treatment significantly decreased the survival rate of WT cells, whereas FABP3 deficiency significantly enhanced cell viability after OGD/R (Figure 3B), suggesting that FABP3 deficiency helps resist OGD/R-induced injury. TUNEL staining results showed that the number of TUNEL-positive cells in WT cells increased significantly after OGD/R, whereas the proportion of TUNEL-positive cells in F3KO cells was significantly reduced (Figure 3C), indicating that FABP3 deficiency alleviated OGD/R-induced apoptosis. Further analysis using TMRE staining showed a significant decrease in TMRE fluorescence intensity in WT cells after OGD/R, indicating a loss of mitochondrial membrane potential. FABP3 deficiency partially restored TMRE signal intensity (Figure 3D), suggesting an alleviation of OGD/R-induced mitochondrial functional impairment. Given the potential involvement of FABP3 in BAX-related mitochondrial apoptosis, levels of BAX and cleaved caspase-3 were analyzed. Western blot results showed that OGD/R significantly increased the BAX/BCL2 ratio and cleaved caspase-3 expression in WT cells, while FABP3 deficiency significantly inhibited this increase (Figure 3G–I). Additionally, the localization relationships among FABP3, activated BAX (6A7), and mitochondria were observed. After OGD/R stimulation, the activated BAX (6A7) was significantly increased in WT cells, exhibiting clear co-localization with FABP3 on mitochondria (Figure 3J). After OGD/R stimulation, the proportion of mitochondria co-localized with both FABP3 and BAX significantly increased (Figure 3K). In F3KO cells, the number of activated BAX localized in mitochondria was significantly reduced (Figure 3L). These findings suggest that under OGD/R conditions, FABP3 may promote BAX activation and its recruitment to mitochondria. Collectively, these results indicate that FABP3 deficiency can alleviate mitochondrial damage, inhibit BAX activation and mitochondrial recruitment, and further attenuate mitochondrial-dependent apoptosis signaling.

### 3.4. Inhibiting BAX Alleviates FABP3 Overexpression-Induced Apoptosis

**Figure 4:**
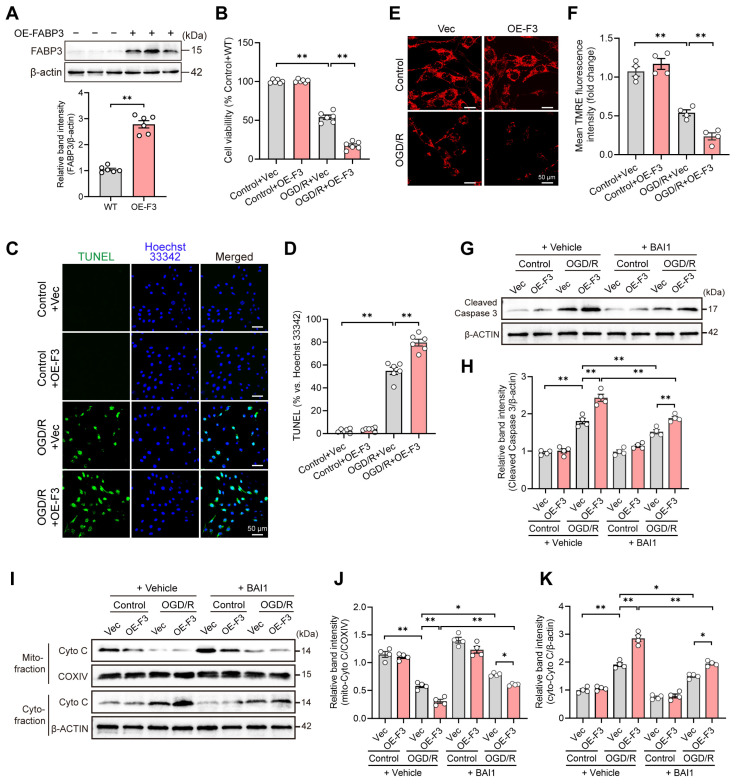
Effects of BAX inhibition on FABP3 overexpression-induced cell apoptosis. (**A**) Representative Western blot images of FABP3 with corresponding quantitative analysis. (**B**) Cell viability measured by the CCK-8 assay. (**C**,**D**) Representative TUNEL staining images (**C**) and quantitative analysis of the percentage of TUNEL-positive cells (**D**). (**E**,**F**) Representative TMRE staining images (**E**) and quantitative analysis of fluorescence intensity (**F**). (**G**,**H**) Representative western blot images of cleaved caspase-3 with quantitative analysis. (**I**–**K**) Representative western blot images of cytochrome c (**I**) and quantitative analysis in the mitochondrial (**J**) and cytosolic (**K**) fractions. COXIV served as the mitochondrial control, and β-actin was used as the cytosolic control. Data are presented as mean ± SEM. * *p* < 0.05, ** *p* < 0.01.

To further validate our findings, we developed a model of FABP3 overexpression in HT22 cells. Western blotting results confirmed a significant increase in intracellular FABP3 protein levels following overexpression (Figure 4A). The CCK-8 assay results demonstrated that FABP3 overexpression exacerbated the reduction in cell viability induced by OGD/R (Figure 4B), suggesting that elevated FABP3 levels enhance cellular susceptibility to OGD/R-induced injury. TUNEL staining revealed that FABP3 overexpression further increased the proportion of TUNEL-positive cells after OGD/R (Figure 4C,D), suggesting that increased FABP3 promoted OGD/R-induced apoptosis. Additionally, TMRE staining indicated a significant decrease in TMRE fluorescence intensity in WT cells following OGD/R stimulation, and FABP3 overexpression further amplified this reduction (Figure 4E,F), suggesting that FABP3 overexpression exacerbates OGD/R-induced mitochondrial dysfunction. To elucidate the involvement of BAX in FABP3-mediated cell damage, we used the BAX inhibitor BAI1. Western blotting results showed that BAI1 treatment significantly reversed the increase in cleaved caspase-3 levels induced by FABP3 overexpression (Figure 4G,H). Furthermore, alterations in cytochrome c distribution indicated that BAI1 treatment significantly inhibited the release of cytochrome c from mitochondria induced by FABP3 overexpression, retaining it in mitochondrial components and reducing its release into cytoplasmic components (Figure 4I–K). These results suggest that inhibiting BAX activation can suppress mitochondrial outer membrane permeability and apoptosis associated with FABP3 overexpression.

### 3.5. FABP3 Promotes Mitochondrial Lipid Overload and Peroxidation to Augment BAX Activation

**Figure 5:**
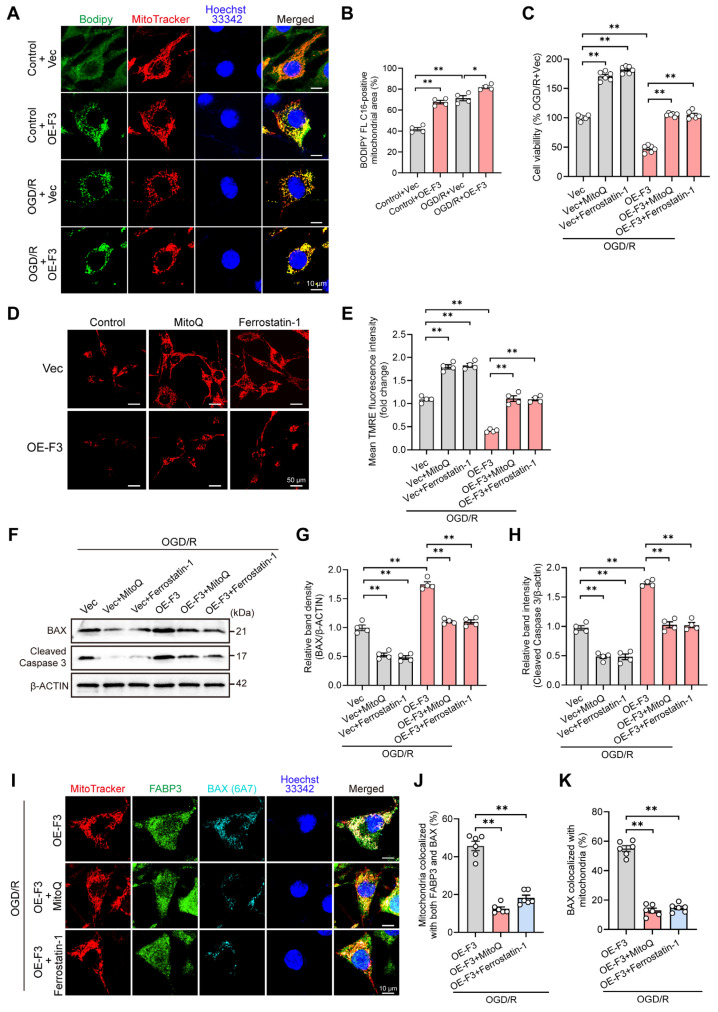
Effects of antioxidants on FABP3 overexpression-induced mitochondrial injury and apoptotic signaling. (**A**) Representative fluorescence images of BODIPY FL C16 (lipid analog, green), MitoTracker (mitochondria, red), and Hoechst 33342 (nuclei, blue). Yellow signals in the merged images indicate the overlap between Bodipy and MitoTracker. (**B**) Quantitative analysis of the percentage of BODIPY FL C16 and MitoTracker co-localized areas relative to the total MitoTracker-positive area. (**C**) Cell viability was measured using the CCK-8 assay. (**D**,**E**) Representative TMRE staining images (**D**) and corresponding quantitative analysis (**E**). (**F**–**H**) Representative Western blotting images of BAX and cleaved caspase-3 (**F**), with quantitative analysis of BAX/β-actin (**G**) and cleaved caspase-3/β-actin (**H**). (**I**–**K**) Representative immunofluorescence images of MitoTracker (mitochondria, red), FABP3 (green), activated BAX (6A7, cyan), and Hoechst 33342 (blue). In the merged images, yellow signals indicate the overlap between MitoTracker and FABP3, whereas white signals indicate the overlap among MitoTracker, FABP3, and BAX (6A7). (**J**) Quantitative analysis of the percentage of FABP3 and BAX co-localized mitochondrial areas relative to the total mitochondrial area. (**K**) Quantitative analysis of the percentage of BAX-colocalized mitochondrial areas relative to the total mitochondrial area. Data are presented as mean ± SEM. * *p* < 0.05, ** *p* < 0.01.

To explore the upstream mechanism underlying the FABP3-induced BAX apoptosis pathway, we focused on FABP3 as a lipid chaperone involved in lipid transport. We assessed alterations in mitochondrial lipid distribution by co-incubating cells with the fluorescent fatty acid analog BODIPY FL C16 and observing its intracellular localization. Under normal conditions, FABP3 overexpression increased mitochondrial lipid accumulation. This phenomenon was further intensified following OGD/R stimulation, as demonstrated by a marked increase in the overlap between BODIPY and MitoTracker signals (Figure 5A). Quantitative analysis corroborated that FABP3 overexpression significantly elevated the proportion of lipids localized in mitochondria under both basal and OGD/R conditions (Figure 5B), suggesting that elevated FABP3 levels promote mitochondrial lipid accumulation, leading to lipid overload. Given that mitochondrial lipid overload is frequently associated with oxidative damage, we employed the mitochondrial-targeted antioxidant MitoQ and the lipid peroxidation inhibitor Ferrostatin-1. CCK-8 assay results showed that under OGD/R conditions, FABP3 overexpression significantly reduced cell viability, whereas treatment with MitoQ or Ferrostatin-1 significantly improved cell viability (Figure 5C). TMRE staining results showed that both MitoQ and Ferrostatin-1 could partially restore the TMRE fluorescence intensity reduced by FABP3 overexpression (Figure 5D,E), indicating that inhibiting mitochondrial oxidative stress or lipid peroxidation can alleviate mitochondrial functional damage induced by FABP3 overexpression. Subsequently, we examined changes in proteins involved in apoptosis. Western blot results showed that treatment with MitoQ or Ferrostatin-1 significantly inhibited the increase in BAX and cleaved caspase-3 caused by FABP3 overexpression (Figure 5F–H). These results suggest that FABP3-mediated mitochondrial damage and apoptosis are closely related to oxidative stress and lipid peroxidation. Furthermore, under OGD/R combined with FABP3 overexpression, the recruitment of activated BAX to mitochondria was significantly increased (Figure 5I). However, following intervention with MitoQ or Ferrostatin-1, the co-localization of FABP3 and BAX in mitochondria decreased (Figure 5J), and mitochondrial BAX signaling activation was attenuated (Figure 5K). These results indicate that FABP3 can enhance BAX activation and recruitment to mitochondria by promoting mitochondrial lipid accumulation and peroxidation, thereby amplifying mitochondrial-dependent apoptosis signals.

## 4. Discussion

In this study, we found that the abnormal elevation of FABP3 under ischemia–reperfusion conditions is not only a stress-related phenomenon but also promotes mitochondrial lipid loading and enhances lipid peroxidation, thereby altering the mitochondrial membrane environment. This process facilitates BAX activation and recruitment to mitochondria, ultimately amplifying mitochondrial-dependent apoptotic signals and exacerbating neuronal damage. These results suggest that the role of FABP3 is not limited to lipid transport, but is more likely to involve linking metabolic imbalance, mitochondrial damage, and the pathological processes of neuronal death.

Our previous research has shown that FABP3 is significantly elevated in the penumbral neurons of the ischemic mouse cortex [24,27], suggesting a close association with ischemic neuronal injury. Based on this observation, we first tested the specific FABP3 inhibitor HY08 and found that it alleviated ischemic brain injury in mice, and this protective effect may involve mitochondrial-related processes [24]. To further clarify the role of FABP3 in ischemic brain injury, we established a stroke model using FABP3 gene-knockout mice. The results indicated that FABP3 deficiency not only reduced cerebral infarction and tissue damage but also improved certain cognitive deficits and spontaneous activity disorders, suggesting that FABP3 deficiency enhances brain tissue tolerance to ischemia–reperfusion injury. Consistent with this, in vitro OGD/R models demonstrated that FABP3 deficiency increased cell viability, reduced apoptosis, and maintained mitochondrial membrane potential; in contrast, FABP3 overexpression exacerbated the injury phenotype on multiple levels. Under basal conditions, FABP3 deletion or overexpression did not markedly alter mitochondrial membrane potential, suggesting that FABP3 is not sufficient to induce overt mitochondrial dysfunction in the absence of ischemia/reoxygenation stress. Rather, its detrimental effect becomes evident when neurons are exposed to OGD/R-induced metabolic and oxidative stress. Combining the pharmacological inhibition results from our previous studies with the genetic intervention results in this study, we conclude that FABP3 is not merely a passive marker after injury but substantively participates in the process of ischemic neuronal injury.

Cerebral ischemic injury is essentially a process that rapidly evolves from metabolic collapse to cellular fate imbalance [30,31], with mitochondria at the center of this transformation [32]. Neurons are highly dependent on mitochondrial homeostasis and are particularly sensitive to mitochondrial damage caused by ischemia–reperfusion. The key factor that advances mitochondrial dysfunction into an irreversible apoptotic program may not solely be the decline in energy production, but rather the disruption of mitochondrial membrane stability and its subsequent chain reactions [33,34]. In this process, the conformational activation of BAX, its recruitment to the mitochondria, and subsequent oligomerization are considered core steps in initiating the intrinsic apoptotic pathway [35]. This study shows that changes in FABP3 levels are closely related to BAX activation, mitochondrial recruitment, cytochrome c release, and the caspase cascade in ischemic neurons. Notably, inhibiting BAX activation partially alleviates mitochondrial damage and reduces the enhancement of apoptotic signals induced by FABP3 overexpression, as evidenced by reduced cytochrome c release and decreased cleaved caspase-3 levels. This indicates that the ischemic neuronal damage mediated by FABP3 is at least partially dependent on the BAX-related mitochondrial apoptotic pathway.

BAX activation is typically regulated by various factors, including BH3-only proteins, oxidative stress, and stress kinases [36]; however, the lipid environment of the outer mitochondrial membrane significantly influences BAX membrane binding, conformational stability, and oligomerization behavior [37,38,39]. In this study, FABP3 was found to enhance mitochondrial lipid loading, whereas the mitochondrial-targeted antioxidant MitoQ reduced the accumulation of activated BAX on mitochondria induced by FABP3 overexpression. This suggests that FABP3 may facilitate a state of increased susceptibility to oxidative damage and pro-apoptotic signals in mitochondria by altering the lipid environment of the mitochondrial membrane and the local redox state. Therefore, the regulation of BAX by FABP3 extends beyond mere expression levels; it is more likely that FABP3 shapes the mitochondrial local oxidative lipid environment, lowering the threshold for BAX recruitment and activation. In other words, the changes mediated by FABP3 are not merely quantitative alterations in lipid levels but may represent qualitative changes in the mitochondrial membrane, providing favorable conditions for BAX insertion into the outer mitochondrial membrane, conformational stability, and oligomerization. In contrast, the lipid peroxidation inhibitor Ferrostatin-1 diminished the accumulation of activated BAX on mitochondria induced by FABP3 overexpression, further supporting the involvement of lipid peroxidation in this process. This is because the reactive aldehyde byproducts generated from lipid peroxidation, such as 4-Hydroxynonenal (4-HNE), can not only disrupt membrane structural stability but may also participate in regulating the activation state of pro-apoptotic proteins [40,41]. Therefore, FABP3-mediated mitochondrial lipid aggregation and its subsequent lipid peroxidation are likely to be significant upstream factors driving BAX-related mitochondrial damage. The regulatory role of FABP3 in ischemic neuronal injury is not limited to general metabolic disorders; rather, it plays a substantial role in the initiation and amplification of mitochondrial apoptotic signaling.

BAX inhibition, mitochondrial antioxidant activity, and lipid peroxidation suppression can partially reverse the damaging effects of FABP3 overexpression. This indicates that FABP3 is not merely a bypass regulatory factor of a terminal event, but rather may occupy a critical position at the intersection of lipid metabolic disorders, oxidative damage, and mitochondrial apoptosis. In the context of stroke, a complex pathological process with multiple interconnected steps, such node-like molecules typically offer greater intervention value than simply blocking a terminal event, as they may simultaneously impact multiple mutually reinforcing damage aspects [42,43,44]. The significance of FABP3 lies in its role, which is not confined to traditional metabolic regulation but rather integrates the abnormal lipid distribution, increased mitochondrial vulnerability, and BAX-dependent apoptosis into a continuous process. Therefore, targeting FABP3 could theoretically yield a more upstream and integrated protective effect than merely inhibiting caspase activation or scavenging reactive oxygen species. In conjunction with our previous observations that FABP3 inhibitors can alleviate brain injury, and with the mechanistic evidence from this study, FABP3 holds potential as a neuroprotective intervention target. Nevertheless, its translational value requires careful evaluation in light of its physiological functions. As a lipid chaperone involved in fatty acid handling and cellular lipid homeostasis, complete or sustained suppression of FABP3 may lead to unintended biological effects. Warren et al. highlighted potential safety implications of FABP inhibition and noted that FABP3 deletion or knockdown may affect cardiac function, metabolism, and cognition [45]. Therefore, therapeutic targeting of FABP3 in ischemic stroke may require an appropriate intervention window, controlled degree of inhibition, cell-type or brain-region selectivity, and preservation of physiological lipid metabolism. Future studies should determine whether transient, partial, or neuron-targeted modulation of FABP3 can provide neuroprotection with an acceptable safety profile.

## 5. Conclusions

In summary, this study indicates that the abnormal upregulation of FABP3 is not merely a concomitant marker of cerebral ischemia–reperfusion injury, but also plays a crucial role in promoting mitochondrial lipid accumulation and lipid peroxidation. This process alters the mitochondrial membrane environment and lowers the threshold for BAX-dependent apoptosis initiation, ultimately exacerbating ischemic neuronal death. Consequently, FABP3 can be regarded as an important node linking lipid imbalance to mitochondrial apoptosis. Targeting FABP3 and its mediated lipid peroxidation–BAX pathway may provide novel research avenues for neuroprotective therapies in ischemic stroke.

## Data Availability

The data that support the findings of this study are available from the corresponding author upon reasonable request.

## Supplementary Materials

The following supporting information can be downloaded at: https://www.mdpi.com/article/10.3390/cells15111003/s1; Figure S1: Baseline cognitive and motor function of mice in each group before MCAO/R surgery; Table S1: Antibodies used for Western blotting and immunofluorescence staining.
