# Supplementary material for: FABP3 Aggravates Cerebral Ischemia–Reperfusion Injury by Promoting Mitochondrial Lipid Accumulation and Enhancing BAX-Dependent Apoptosis
Source: Cells. 2026 May 29;15(11):1003. doi: 10.3390/cells15111003 (PMC13256585; doi:10.3390/cells15111003)
Supplement: Supplementary file 1 [file cells-15-01003-s001.zip › cells-4260792-supplementary.pdf]

Supporting Information

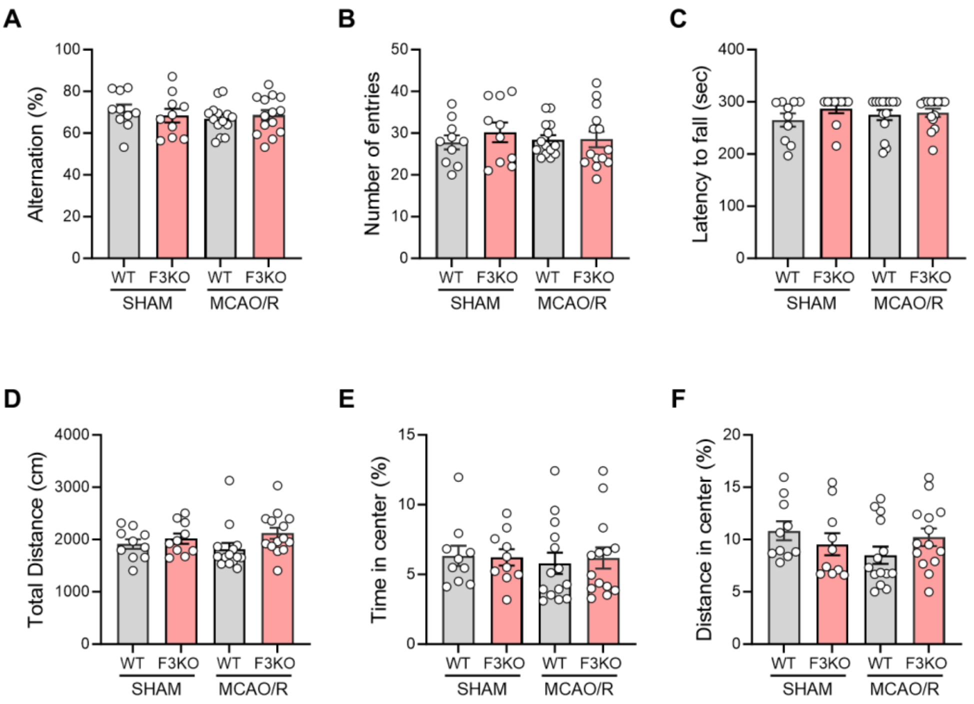

Supplemental Figure S1. Baseline cognitive and motor function of mice in each group before ischemia. (A–B) Spontaneous alternation rate (A) and total number of arm entries (B) in the Y-maze test. (C) Fall latency in the rotarod test. (D–F) Total distance traveled (D), percentage of time spent in the central region (E), and percentage of distance traveled in the central region (F) in the open field test. Data are expressed as mean ±SEM (n = 10~14).

Table S1: Antibodies used for western blotting and immunofluorescence staining

Designation	Source	Identifiers	Dilution ratio	RRID
FABP3	Proteintech	10676-1-AP	1:1000 (WB)/ 1:200 (IF)	AB_2102309
FABP3	Proteintech	60280-1-Ig	1:500 (IF)	AB_2881398
Cleaved Caspase-3	Proteintech	66470-2-Ig	1:2000 (WB)	AB_2876892
β-actin	Proteintech	66009-1-Ig	1:5000 (WB)	AB_2687938
Cytochrome c	Proteintech	66264-1-Ig	1:5000 (WB)	AB_2716798
COXIV	Proteintech	11242-1-AP	1:5000 (WB)	AB_2085278
TOM20	Proteintech	11802-1-AP	1:5000 (WB)	AB_2207530
BAX	Proteintech	60267-1-Ig	1:5000 (WB)	AB_2848213
BAX (6A7)	Santa Cruz	sc-23959	1:100 (IF)	AB_626728
Bcl-2	Proteintech	60178-1-Ig	1:1000 (WB)	AB_10734459

WB: Western Blot; IF: Immunofluorescence
